# Inhibition of GPR158 by microRNA-449a suppresses neural lineage of glioma stem/progenitor cells and correlates with higher glioma grades

**DOI:** 10.1038/s41388-018-0277-1

**Published:** 2018-05-03

**Authors:** Ningning Li, Ying Zhang, Kastytis Sidlauskas, Matthew Ellis, Ian Evans, Paul Frankel, Joanne Lau, Tedani El-Hassan, Loredana Guglielmi, Jessica Broni, Angela Richard-Loendt, Sebastian Brandner

**Affiliations:** 1 Department of Neurodegeneration, Institute of Neurology, University College London, Queen Square, London, WC1N 3BG UK; 2 The Seventh Affiliated Hospital of Sun Yat-sen University, Shenzhen, 518107 China; 3 Division of Medicine, University College London, University Street, London, WC1E 6JF UK; 4 Division of Neuropathology, the National Hospital for Neurology and Neurosurgery, University College London Hospitals NHS Foundation Trust Queen Square, London, WC1N 3BG UK; 5 Blizard Institute, Barts and the London School of Medicine and Dentistry, Queen Mary University of London, 4 Newark Street, London, E1 2AT UK; 6 UCL IQPath laboratory, Institute of Neurology, University College London, Queen Square, London, WC1N 3BG UK

## Abstract

To identify biomarkers for glioma growth, invasion and progression, we used a candidate gene approach in mouse models with two complementary brain tumour phenotypes, developing either slow-growing, diffusely infiltrating gliomas or highly proliferative, non-invasive primitive neural tumours. In a microRNA screen we first identified microRNA-449a as most significantly differentially expressed between these two tumour types. miR-449a has a target dependent effect, inhibiting cell growth and migration by downregulation of CCND1 and suppressing neural phenotypes by inhibition of G protein coupled-receptor (GPR) 158. GPR158 promotes glioma stem cell differentiation and induces apoptosis and is highest expressed in the cerebral cortex and in oligodendrogliomas, lower in IDH mutant astrocytomas and lowest in the most malignant form of glioma, IDH wild-type glioblastoma. The correlation of GPR158 expression with molecular subtypes, patient survival and therapy response suggests a possible role of GPR158 as prognostic biomarker in human gliomas.

## Introduction

The prognostication of human gliomas has seen significant changes over the last 10 years. The identification of mutations in two isocitrate dehydrogenase genes, IDH1 and IDH2, in gliomas [1] was a major discovery, leading to a biomarker-defined glioma classification, IDH and ATRX-mutant astrocytomas and glioblastomas and IDH-mutant 1p/19q codeleted oligodendrogliomas [2]. The clinical value of molecular subtyping of IDH wild-type glioblastoma instead had limited clinical impact [3, 4]. The only prognostic biomarker in GBM is the methylation of MGMT but is has no diagnostic value [5].

**Fig. 1:**
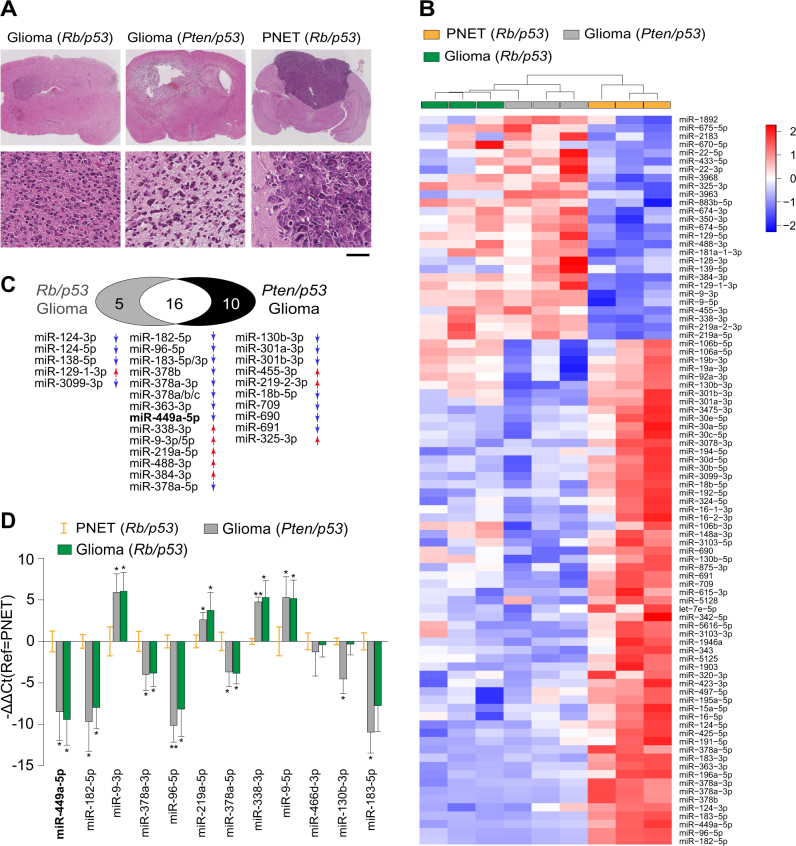
Identification and validation of miRNAs, differentially expressed (DEmiRs) between glioma and PNET. **a** Representative histology of *Rb/p53*-mutant glioma (left) and PNET (right), and *Pten/p53*-mutant glioma (centre). Scale bar corresponds to 1 mm in the upper row (overview) and 50 µm in the lower row (detail). It shows the infiltrative growth of gliomas (columns 1&2) and the demarcated margins of the hyper-cellular PNET (column 3). **b** Heat map of DEmiRs between *Pten/p53*-glioma, *Rb/p53*-glioma, and *Rb/p53*-PNET (one-way ANOVA, *p* < 0.05). Unsupervised clustering analysis was performed on R. **c** Venn diagram of DEmiRs between *Rb/p53* and *Pten/p53*-mutant gliomas. Upregulation indicated with red, and down regulation with blue arrows next to the miRNAs. **d** Validation of DEmiRs by RT-qPCR. In each panel, *Rb/p53-*PNETs are set as 'calibrator' sample (orange error bars), and the y-axis represents log2 fold change (−ΔΔCp) with samples as indicated in the figure legend. The bars are means ± s.e.m. (Student**’**s *t*-test; *FDR < 0.05; **FDR < 0.01)

To identify additional biomarkers of diagnostic and/or prognostic value, we used a mouse model of intrinsic brain tumours generated by Cre-mediated inactivation of *Pten*^*lox/lox*^ and *p53*^*lox/lox*^ genes or of *Rb*^*lox/lox*^ and *p53*^*lox/lox*^ genes in the neurogenic cell population of the subventricular zone (SVZ) of the brain, previously in-depth molecularly characterized [6, 7]. Mice with tumours mutant for *Pten* and *p53* (in short *Pten/p53*) develop diffusely infiltrative high grade gliomas (Fig. 1a) [8–10] with an expression profile of the TCGA classical GBM [4] or Phillips proneural [11]. Cre-mediated recombination of the *Rb* and *p53* genes (in short *Rb/p53*) in the SVZ, gives rise to poorly differentiated, well-demarcated tumours with a primitive neural phenotype and less frequently also to gliomas (Fig. 1a) [6–8]. These tumours, previously described as primitive neuroectodermal tumours (PNET), have an expression profile corresponding to a malignant childhood tumour, atypical teratoid/rhabdoid tumour (AT/RT) [6, 12]. We chose to compare these two models of intrinsic brain tumours with their distinctive morphology, survival rates and transcriptomic profiles, to identify mechanisms controlling growth, differentiation and tumour invasion, and corresponding biomarkers in human gliomas.

First we set out to discover differentially expressed micro-RNAs (miRNAs, miR), small non-coding RNAs regulating gene expression at post-transcriptional level by binding to the 3′ untranslated region (UTR) of the targeted mRNA for degradation [13]. miRNAs participate in controlling many biological processes, such as cell cycle, apoptosis, stem cell differentiation, and immune responses [14], and they also play a key role in the differentiation and maintenance of tissue identity [15]. In our screen, miR-449a was amongst the most significantly differentially regulated. It was selected for subsequent analysis as a tumour suppressive role of miR-449a has been suggested in a number of malignancies [16–19], most notably in prostate cancer by targeting classical proto-oncogenes CCND1 [20], c-Myc [21] and HDAC-1 [22]. miR-449a is a direct transcriptional target of E2F1, negatively regulating pRb-E2F1 activity by CDK6 and CDC25A [23]. A role of miR-449a targeting MYC-associated zinc finger proteins has been suggested in glioblastoma [17]. In contrast, during neural development miR-449a has a different regulatory role. It is expressed highest during the proliferative phase of embryonic neurogenesis [24] and is essential for the production of intermediate progenitors during cortical development [25, 26]. Amongst the established targets of the miR-449 family are E2F1, CDK6, CCND1 and BCL2 [20, 23, 27]. miR-449a targets a site in the 3′ UTR of the CCND1 transcript, and miR-449a significantly reduces Cyclin D1 protein in PC-3 cells [20]. Here, we identify a new target of miR-449a, the G-protein coupled receptor 158 (GPR158), a member of a large group of cell surface proteins exerting a range of diverse cellular functions. GPR158 and two others, GPR156 and GPR179 belong into the gamma-aminobutyric acid receptor branch of the GPCR glutamate family (Group III), containing 7 orphan receptors [28]. The first identified roles of GPR158 were those of a plasma membrane scaffold protein in retinal bipolar neurons and [29], and the expression in trabecular meshwork cells in the eye’s aqueous outflow pathways, contributing to the pathophysiology of steroid-induced ocular hypertension and glaucoma [30]. It has relevance to prostate cancer growth and progression [31], and a role in lung cancer outcome was identified [32], thus presenting a potential relevant link to our findings in brain tumours. Here we show a target dependent effect of miR-449a, inhibiting growth and migration by downregulating CCND1 and suppressing neural differentiation by inhibiting GPR158. In human gliomas, high levels of miR-449a and low expression of GPR158 are associated with higher malignancy and poorer survival.

## Results

### miR-449a is significantly differentially expressed between experimental gliomas and primitive neural tumours

To identify genes that are differentially expressed (DE) between gliomas and PNET (Fig. 1a) we performed miRNA microarrays. Unsupervised hierarchical clustering identified 89 differentially expressed miRNAs (DE-miRs) between gliomas of both genotypes (*Pten/p53* and *Rb/p53)*, and PNET’s (*Rb/p53)* (Fig. 1b, Supplementary Table 1). Twenty miRs were differentially expressed between gliomas (*Pten/p53*) and PNETs (*Rb/p53*) (Supplementary Table 1). Twenty-six significantly DE-miRs were identified between gliomas (*Pten/p53*) and PNETs (*Rb/p53*), and 21 top DE-miRs between *Rb/p53* glioma and PNETs (Fig. 1c). We found a high degree of overlap with 16 miRNAs co-existing in both DE-miR groups. Reverse transcription (RT)-quantitative PCR (RT-qPCR) reduced the group to 9 DE-miRs between gliomas and PNETs (Fig. 1d; Supplementary Table 1), and of those, miR-449a was most significantly differentially expressed (Fig. 1d). Gene ontology analysis of these nine miRNAs showed an association with neurogenesis and cell migration (Supplementary Table 2). miR-449a is enriched in astrocytes [33], whereas miR-219 and miR-338 are essential for oligodendrocyte differentiation [15]. Considering that miR-449a is involved in the regulatory network of *RB* and *P53* [23, 34], it was a promising candidate and most likely relevant to the brain tumour phenotype.

### **miR-449a directly targets*Ccnd1*and*Gpr158***

**Fig. 2:**
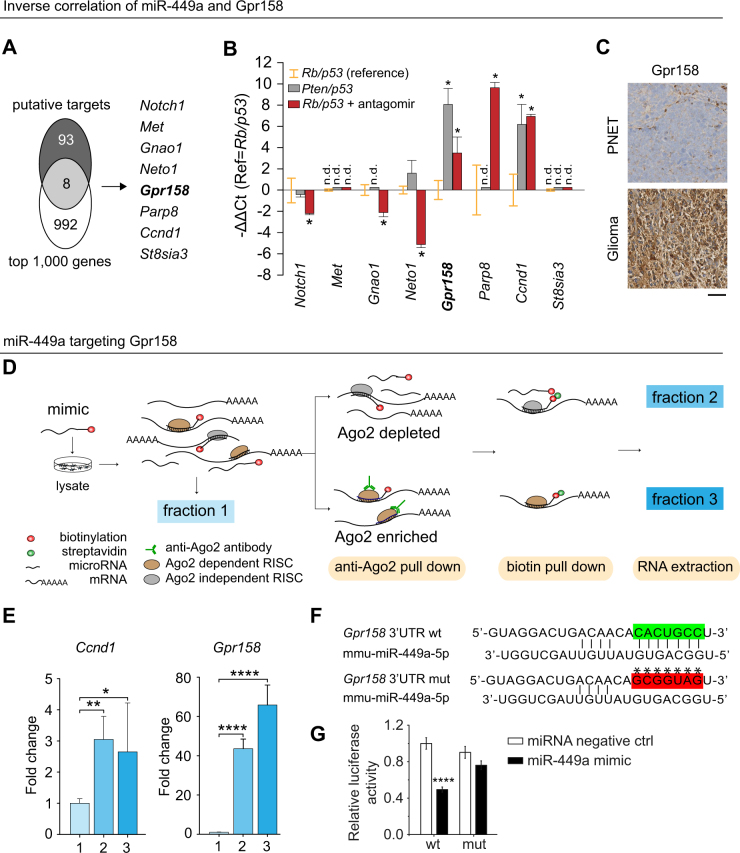
Identification of *Gpr158* as a direct target of miR-449a. **a** Venn diagram with eight candidate genes emanating from 101 in silico putative targets and 1000 down-regulated genes in experimental PNETs compared with gliomas by analysis of exon expression array. **b** Candidate gene expression level is validated by RT-qPCR in *Rb/p53* (orange error bars), *Rb/p53*^ant^ (red bars) and *Pten/p53* cells (grey bars). Most differentially expressed *Ccnd1* and *Gpr158* are further analysed, as their expression is similar in *Rb/p53*^ant^ and *Pten/p53* cells, but significantly higher than in *Rb/p53* cells. **c** IHC staining shows that Gpr158 expression is minimal in miR-449 highly expressing PNETs, but strong in miR-449 low expressing gliomas. Scale bar 50 µm. **d** Schematic illustration of Ago2 and biotin double pull-down assay for assessment of miRNA-mRNA binding. Commercial synthetic miR-449a mimics are transfected into neural stem cells, and Ago2 immunoprecipitation is carried out to confirm that miRNA-mRNA binding is RISC dependent. Fraction 1 represents the input RNA, fraction 2 the Ago2 depleted fraction, i.e, miRNA and mRNA unbound to Ago2. Fraction 3 represents miRNA449a-mRNA complex bound to Ago2, representing the degradation complex RISC. These fractions were then tested for the enrichment of *Gpr158* and *Ccnd1* transcripts: **e** Enrichment of *Ccnd1* and *Gpr158* is measured after pull-down using RT-qPCR. The x axis shows the fraction as described in (**d**). There is a highly significant enrichment in fraction 3 (Ago2-dependent miR-449a –*Gpr158* complex) indicating direct interaction. **f** miR-449a binding sequence in the 3′ UTR of *Gpr158*. A mutation of the 3**’**UTR of *Gpr158* generated in the site complementary to the seed region of miR-449a. *Indicates the mutant nucleotides. **g** miR-449a directly targets *Gpr158* by interacting with its 3′ UTR. Relative luciferase activity (normalized to control) of BTSCs transfected with pMIR-Gpr158-3′ UTR-wt or pMIR-Gpr158-3′ UTR-mut, and co-transfected with miRNA negative control or miR-449a mimics. This suggests a significant miR-449a mediated downregulation of *Gpr158*, which is not seen in the mutant control. All figures: *p ≤ 0.05; **p ≤ 0.01; ***p ≤ 0.001; ****p ≤ 0.0001 (Student**’**s *t*-test). Each bar represents mean ± sd

miR-449a targets were identified with TargetScan 7.1, resulting in a list of 101 putative targets with conserved binding sites (Fig. 2a; Supplementary Table 1). To identify DE genes between the two tumour types, we retrieved the top 1000 DE-genes ranked by logarithmic fold change (glioma/PNET) from our published Exon Microarray dataset (GSE42515), and matched them against the 101 putative targets, following permissive filtration criteria: (i) miR-449a is highly expressed in PNETs, expecting downregulated targets; (ii) DE-genes with *p* > 0.05 were also considered to minimize false negative calls. Eight genes were selected (*Notch1, Met, Gnao1, Meto1, Gpr158, Parp8, Ccnd1 and St8sia3*) (Fig. 2a,b). To identify candidates regulated by miR-449a, we analysed *Rb/p53* (miR-449a^high^), *Pten/p53* (miR-449a^low^), and *Rb/p53*^antagomir^ mBTSCs (miR-449a^low^), and selected those targets which were upregulated in miR-449a^low^ mBTSCs. Quantification confirmed that only *Gpr158* and *Ccnd1*, but not the other six genes, were correspondingly regulated in both *Pten/p53* and *Rb/p53*^antagomir^ cells, compared to the baseline of *Rb/p53* cells (Fig. 2b). *Ccnd1* and *Gpr158* carry conserved miR-449a binding sites within their 3′ UTR [20], (Fig. 2f). In keeping, primary *Rb/p53* brain tumours (PNET) express low, and *Pten/p53* gliomas high Gpr158 levels (Fig. 2c).

We then confirmed a functional link between miR-449a and its target *Gpr158* by two functionally independent approaches: a modified hybrid Argonaute 2 (AGO2) pulldown assay and a luciferase reporter assay. The AGO2 assay [35, 36] (Fig. 2d) uses an established miR-449a target, CCND1, as positive control [20]. After double pull-downs of AGO2 and biotin-labelled miR-449a mimics in tandem, the relative enrichment of each fragment compared to input RNA was measured by RT-qPCR (Fig. 2e, number corresponds to the fraction in Fig. 2d). We confirmed that the degradation of *Gpr158* mRNA by miR-449a was dependent of RNA-induced silencing complex [37] (Fig. 2e), demonstrating direct regulation of *Gpr158* expression by miR-449a. The luciferase assay confirmed physical binding of miR-449a to the *Gpr158* 3′ UTR and to locate the conserved binding sequence within the 3′ UTR of *Gpr158*. Luciferase reporter plasmids containing wild-type (*pMIR-Gpr158-3*′ *UTR-wt*) and mutant (*pMIR-Gpr158-3*′ *UTR-mut*) sequence complementary to the seed region of miR-449a were generated (Fig. 2f). Mir-449a mimics significantly reduced luciferase activity under a wild-type 3′ UTR (*p* < 0.001), which was rescued by substituting the reporter plasmid with a mutant seed sequence (Fig. 2g), indicating that the mutated 3′ UTR of *Gpr158* reduced miR-449a binding.

### miR-449a inhibits brain tumour stem cell self-renewal, proliferation and migration in vitro

**Fig. 3:**
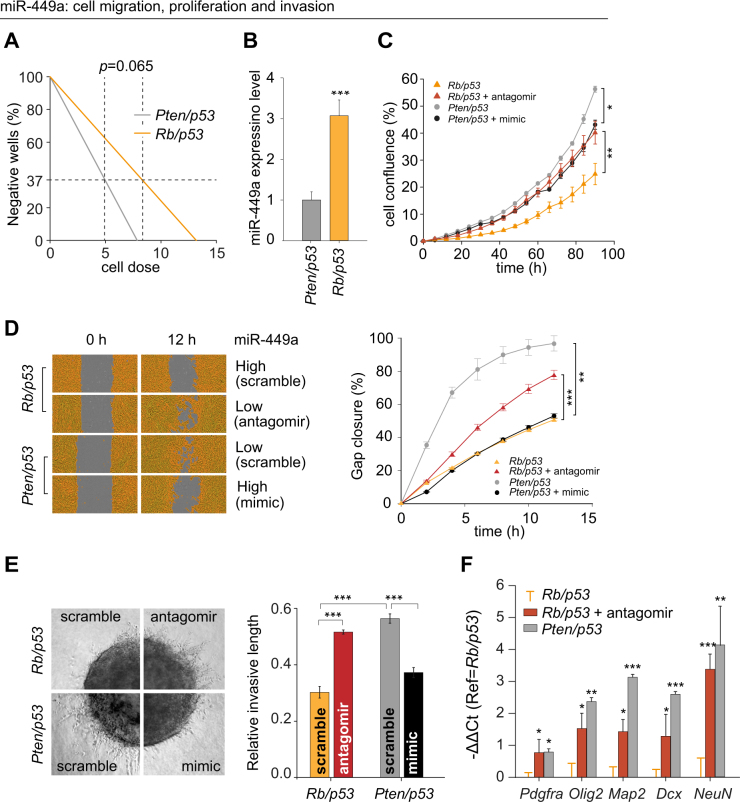
miR-449a inhibits cell proliferation and migration of BTSC in vitro. **a** Analysis of the stem cell frequency of *Pten/p53* and *Rb/p53* BTSCs using the extreme limiting dilution assay. Negative wells were counted 7 days post-seeding. Frequency of sphere-forming cells: *Pten/p53:* 1/4.96; *Rb/p53:* 1/8.32, *n* = 12, *p* = 0.065, indicating lower rate of self-renewal of *Rb/p53* cells. **b** High levels of miR-449a in *Rb/p53* mBTSC compared to *Pten/p53* BTSC (*p* < 0.001), normalized for *Pten/p53*. **c** miR-449a reduces cell proliferation (confluence assay): *Pten/p53* (miR-449a^low^) BTSC grow faster than *Rb/p53* (miR-449a^high^) mBTSC. Treatment of *Pten/p53* BTSCs with miR-449a mimic reduces their proliferation, whilst antagomir-treated *Rb/p53* (*Rb/p53*^ant^) BTSC show increased proliferation, compared to scrambled-treated controls, respectively. **d** The gap closure assay shows the cell migration over 12 h into an artificially generated cell-free space. miR-449a^low^ cells (i.e. *Rb/p53*^ant^ and *Pten/p53*^scramble^) migrate faster and almost close the gap after 12 h; whereas miR-449a^high^ cells (i.e., *Rb/p53*^scramble^ and *Pten/p53*^mimics^) hardly advance into the gap. Recording of multiple time points during the 12 h period visualizes the dynamics of migration and confirms a statistically significant difference in the live cell imaging assay (right figure part). **e** Quantification of the invasion of a mass of 5000 cells into 3D collagen. The left figure part shows an example of the outgrowth of processes from spheres. miR-449a^low^ cells (i.e., *Rb/p53*^ant^ (top right) and *Pten/p53*^scramble^ (bottom left)) show substantial outgrowth into the matrix. In contrast, miR-449a^high^ cells (i.e., *Rb/p53*^scramble^ (top left) and *Pten/p53*^mimics^ (bottom right)) hardly advance into the matrix (left panel). The right figure part shows quantification with Image J, confirming the statistically significant difference (*n* = 12). **f** RT-qPCR analysis of differentiation markers in BTSC shows a proneural gene expression pattern in *Pten/p53* and in antagomir-treated *Rb/p53* BTSCs compared to the baseline of untreated *Rb/p53* BTSC (orange error bars). Inhibition of miR-449a (i.e., *Rb/p53*^ant^, red bars) resulted in the shift of basal expression profile of *Rb/p53* cells towards that of *Pten/p53* BTSCs (grey bars)

To characterize the role of miR-449a in brain tumour stem cells (BTSC), murine (m)BTSC were derived from SVZ stem/progenitor cells of naïve *Pten/p53* or *Rb/p53* mice, recombined in vitro [7] and expanded in EGF and FGF-enriched serum-free stem cell medium [38]. An in vitro extreme limiting dilution assay [39] showed a tendency (*p* = 0.065) of reduced self-renewal of *Rb/p53* BTSC (miR-449a^high^), compared to *Pten/p53* cells (miR-449a^low^) (Figs. 3a, b). In a proliferation assay, *Pten/p53* cells grew faster (Fig. 3c) and in a gap closure assay they moved faster than *Rb/p53* cells into an artificially introduced gap (Fig. 3d). To test if these were miR-449a-mediated effects, miR-449a antagomir or mimics were introduced into mBTSCs. Transient inhibition of miR-449a with antagomir in *Rb/p53* mBTSCs significantly increased their proliferation and migration, consistent with previous reports in cell lines derived from hepatocellular carcinoma [16], colon cancer [27], or prostate cancer [22], whilst the opposite effect was seen when a miR-449a mimic was introduced into *Pten/p53* cells (Figs. 3c, d). In a 3D collagen matrix tumour invasion assay, inhibition of miR-449a expression in *Rb/p53* cells increased cell migration, whereas miR-449 mimics in *Pten/p53* cells slowed down migration (Fig. 3e), consistent with findings in hepatocellular carcinoma cells [16]. In conclusion, in stem cell medium, miR-449a^high^ mBTSC grow slower than miR-449a^low^ mBTSC, suggesting a suppressive role of miR-449a on proliferation, migration and invasion.

### miR-449a targets *Ccnd1*, inhibiting proliferation and migration of BTSC in vitro

**Fig. 4:**
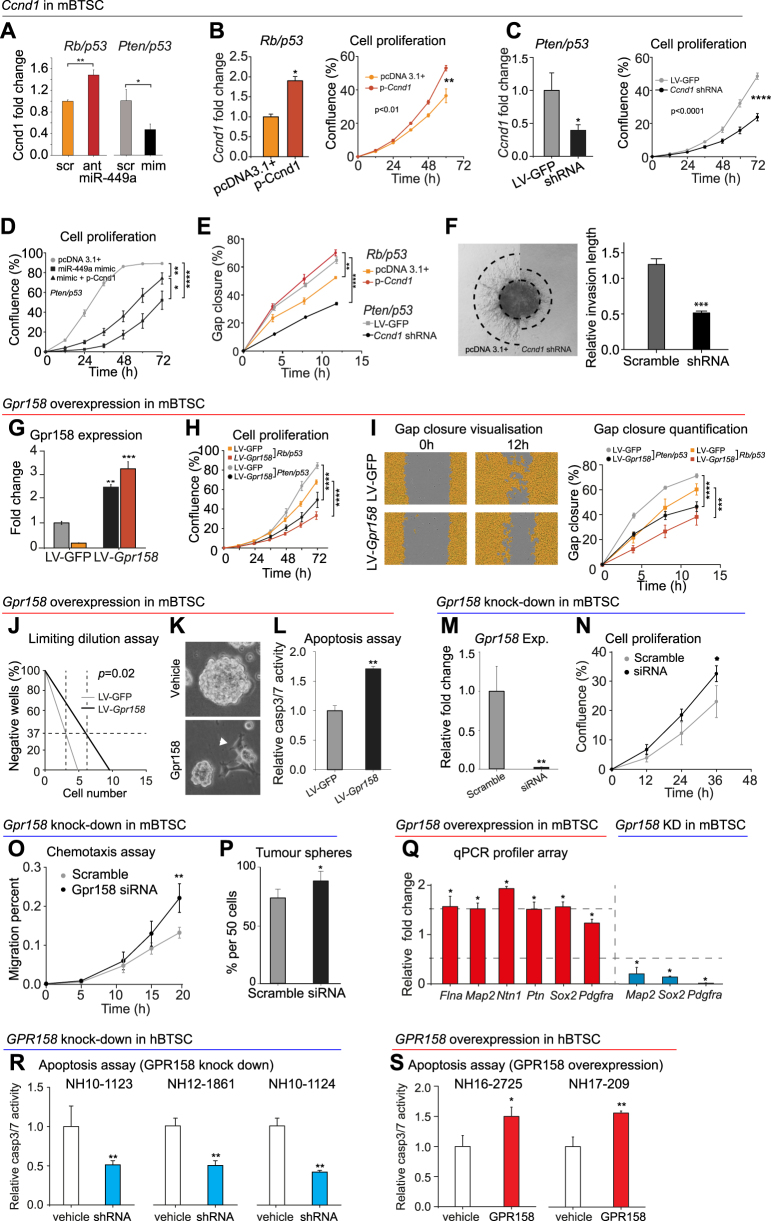
miR-449a reduces cell proliferation and migration by suppressing Ccnd1, and neural phenotypes and apoptosis by suppressing GPR158 in mBTSC. **a** miR-449a reduces Ccnd1 levels in mBTSC: miR-449a antagomir (ant) treatment of mi449a^high^ *Rb/p53* mBTSC restores *Ccnd1* expression, and conversely miR-449a mimic (mim) treatment of *Pten/p53* miR-449a^low^ mBTSC reduces Ccnd1 expression. Scr = scramble (**b**) Transient transfection of *Rb/p53* mBTSC with a *Ccnd1* expression vector results in twofold overexpression of Ccnd1 and increased cell proliferation, and **c** knockdown decreases it. **d** Forced *Ccnd1* overexpression (transfection) antagonises miR-449a -mediated inhibition of cell proliferation. Top curve (grey) baseline *Pten/p53* (miR-449a^low^), bottom curve miR-449a knockdown, and middle curve miR-449a kd + *Ccnd1* restore. **e** Ccnd1 accelerates cell proliferation in a confluence assay: *Pten/p53* cells (grey) grow faster than *Rb/p53* cells (orange). *Ccnd1* overexpression increases proliferation of *Rb/p53* cells (red) which now proliferate faster than *Pten/p53* cells. These grow slower than untransfected cells *Rb/p53* cells (orange) when Ccnd1 is inhibited (black). **f** Inhibition of *Ccnd1* reduces outgrowth of tumour sphere processes, demonstrating the role of Ccnd1 in cell proliferation and migration. G-S, effects of overexpression or inhibition of Gpr158 in *Rb/p53* or *Pten/p53* mBTSC (**g**) Gpr158 levels in naïve and Gpr158 transfected *Rb/p53* or *Pten/p53* mBTSC. **h** Gpr158 downregulates cell proliferation, **i** cell migration and **j** self-renewal proportionally in *Rb/p53* or *Pten/p53* mBTSC. A 2-fold decrease of tumour sphere forming cells was observed upon Gpr158 overexpression. BTSCs stably expressing Gpr158 = 1/6; BTSCs control = 1/3; i.e., requiring the presence of 3 cells to form 1 neurosphere in controls, vs. 6 cells to form a sphere in Gpr158 overexpressors (*p* = 0.02) *n* = 12; *p* = 0.02. **k** Suspension culture of mBTSCs in serum-free medium. Upon stable expression of Gpr158, mBTSC attach to the surface of the cell culture well, change morphology and involute/grow slower. **l** The Caspase-3/7 activity assay indicates that Gpr158 significantly increases apoptosis in mBTSC. **m** Knock-down of Gpr158 using siRNA in mouse BTSCs, confirmation of abolition of Gpr158 mRNA expression. Down-regulation of *Gpr158* promotes cell proliferation (**n**), migration (**o**) and tumour sphere forming ability (**p**). **q** Stable expression of *Gpr158* significantly upregulates expression of neural genes, assessed in a mouse neurogenesis qPCR profiler array, while siRNA knock-down of *Gpr158* significantly reduces the expression of *Map2, Sox2* and *Pdgfra*. **r** Stable knock-down of *GPR158* in three human GBM primary cell lines cultured in serum-free medium, containing hBTSC reduces BTSC apoptosis. **s** Overexpression of *GPR158* significantly increases apoptosis of human GBM primary cells (hBTSC). All figures: *p ≤ 0.05; **p ≤ 0.01; ***p ≤ 0.001; ****p ≤ 0.0001 (Student’s *t*-test). Each assay was performed at least twice

First we confirmed the known effect of miR-449a to downregulate *Ccnd1*. In keeping, treatment of *Rb/p53* mBTSC (miR-449a^high^) with miR-449a antagomir rescued (i.e., increased) *Ccnd1* expression, whilst treatment of *Pten/p53* mBTSC (miR-449a^low^) with miR-449a mimic reduced *Ccnd1* expression (Fig. 4a). This effect can be antagonised by overexpressing *Ccnd1* in *Rb/p53* mBTSC, increasing proliferation (Fig. 4b), or by *Ccnd1* siRNA treatment of *Pten/p53* mBTSC, reducing proliferation (Fig. 4c), consistent with previous reports [27]. Co-transfection of *Pten/p53* mBTSC with mir-449a mimic and *Ccnd1* expression vector restored the reduced proliferation of cells treated with mir-449a mimic alone (Fig. 4d). Likewise Ccnd1 overexpression increases migration of *Rb/p53* cells, and inversely inhibition in *Pten/p53* cells inhibits cell migration in a gap closure assay (Fig. 4e). In keeping the ability of *Pten/p53* (miR-449a^low^; Ccnd1^high^) mBTSC to invade a matrigel matrix is antagonised by Ccnd1 shRNA (Fig. 4f).

### The miR-449a target *Gpr158* promotes differentiation and apoptosis, and inhibits proliferation and migration of BTSC in vitro

Next we characterized the effects of miR-449a action on *Gpr158* in vitro (Fig. 4). Expression levels of GPR158 are highest in murine neural stem/precursors cells (mNSPC), lower in *Pten/p53* and virtually undetectable in *Rb/p53* mBTSC (Supplementary figure 1A). Unexpectedly, overexpression of *Gpr158* in *Rb/p53 and Pten/p53* mBTSC, resulted in slower growth of both cell lines, whereby *Pten/p53* cells always proliferated and migrated faster than *Rb/p53* cells (Figs. 4g-i), i.e., Gpr158 further reduces proliferation and migration. This is seemingly incompatible with the finding that miR-449a inhibits Gpr158, as both miR-449a^high^ *Rb/p53* and miR-449a^low^ *Pten/p53* cells show further reduction of proliferation, i.e., in the same direction as miR-449a treatment. However, when we explored if Gpr158 regulates the ability of BTSC to form tumour spheres, we found in an extreme limiting dilution assay [39] a significant reduction of sphere forming cells upon Gpr158 overexpression (Fig. 4j). Gpr158 overexpressing BTSC were smaller and tended to attach and differentiate (Fig. 4k), suggesting a role of Gpr158 to induce neural differentiation, and the potential to override miR-449a effects. Indeed siRNA knock-down of *Gpr158* in naïve, adherently growing murine neural stem/progenitor cells (mNSPC) (Supplementary Figure 1B) showed global reduction of expression levels of the majority of genes associated with neural differentiation/neurogenesis (Supplementary Figure 1C), of which *Nrp1*, *S100α6* and *Tnr* were significant. Knockdown of *Gpr158* in *Pten/p53* mBTSCs (Fig. 4m) increases cell migration (Fig. 4o), and sphere formation (Fig. 4p). The role of GPR158 to induce neural marker expression, was quantified in a qRT-PCR profiler assay. There is significant up-regulation of the proneural markers *Map2*, *Sox2*, and *Pdgfra* [4], and of three extracellular matrix-associated genes *Filamin A (Flna), Netrin 1 (Ntn1)*, and *Pleiotrophin (Ptn)*, and downregulation upon *Gpr158* knock-down (Fig. 4q).

Neural differentiation of stem and progenitor cells is associated with apoptotic cell death [40], and in keeping, overexpression of *Gpr158* in mBTSC resulted in significant induction of caspase-3/7 activities (Fig. 4l), whilst *Gpr158* siRNA knockdown increased proliferation (*p* < 0.05, Figs. 4m, n), and migration (Fig. 4o), and there was increased sphere formation (Fig. 4p). Consistent with this observation, also *GPR158* knockdown in three human GBM primary cultures significantly reduced apoptosis (Fig. 4r), and in keeping, overexpression of GPR158 in two human GBM primary cultures induced apoptosis (Fig. 4s). Baseline levels of a selection of glioma cell lines is shown in Supplementary Figure 3G.

In conclusion, increase of GPR158 expression in mBTSC and hBTSC reduces proliferation, migration and cancer stem cell formation, upregulation of proneural markers and induction of apoptosis whilst downregulation of GPR158 has the opposite effect. We identified that miR-449a has distinct, target-dependent (i.e., CCND1 and GPR158) effects on cellular growth, migration and differentiation.

### BTSC differentiation induced by GPR158 is antagonised by miR-449a

**Fig. 5:**
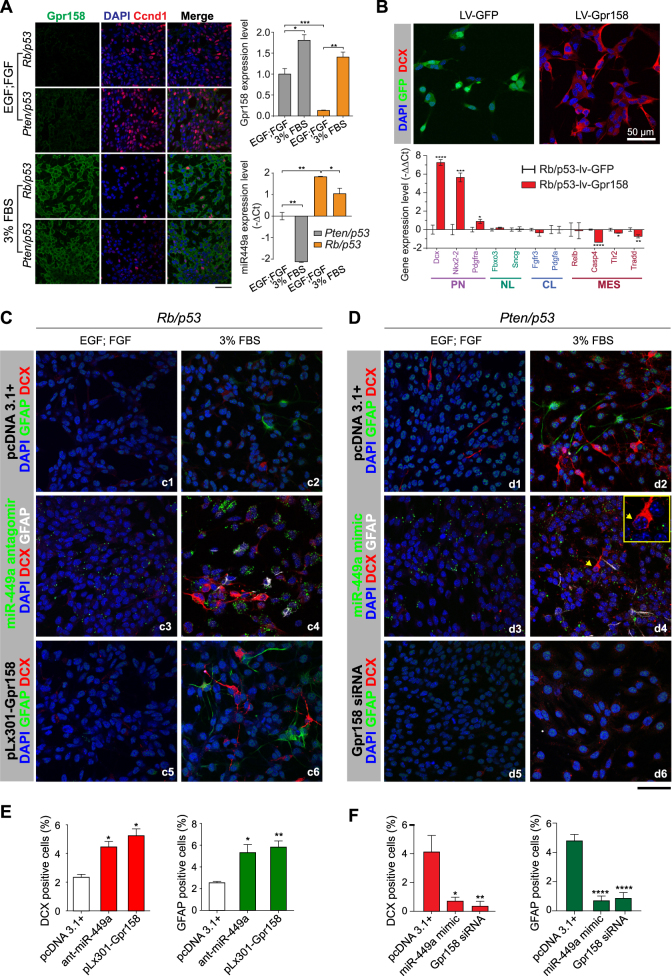
miR-449a antagonises Gpr158-induced neural differentiation. **a** Induction of neural differentiation with 3% FBS increases Gpr158 and reduces miR-449a expression. Ccnd1 expression in EGF, FGF-enriched stem cell medium (upper part) is suppressed in miR-449a^high^ *Rb/p53* with fewer nuclei labelled, and the overall expression is strongly reduces upon growth in 3% FBS supplemented medium. Quantification of miR-449a and Gpr158 expression in these cells on the right. **b** Stable overexpression of Gpr158 antagonises miR-449a and promotes neural differentiation in miR-449a^high^ *Rb/p53* mBTSC. Upper panel, cells transformed with lentivirus expressing GFP or an Gpr158 expressing lentivirus, stained for doublecortin to label postmitotic neural progenitor cells and early immature neurons in serum-free, EGF and FGF enriched stem cell medium. RT-PCR profiling shows expression of 'proneural' and downregulation of 'mesenchymal' genes upon Gpr158 expression (**c**) Knock-down of miR-449a or overexpression Gpr158 induce glial and neural marker expression. *Rb/p53* mBTSC (miR-449a^high^), were either transfected with vehicle (pcDNA 3.1+, c1), miR-449a antagomir (GFP labelled, c3) or the lentivirus pLX301-Gpr158 (c5). Following exposure to 3% FBS for 48 h (right panel, c2, 4, 6), only miR-449a^low^ cells (c4) or increase of GPR158 levels (b6) but not vector-only transfected controls (b2) show increased GFAP and DCX positive mBTSC. **d** The opposite result is seen with *Pten/p53* mBTSC (miR-449a^low^) are treated with miR-449a mimic or Gpr158 siRNA. In EGF, FGF enriched, serum free medium only rare DCX positive mBTSC are seen (d1). Exposure to 3%FBS enriches in GFAP or DCX expressing mBTSC (d2). Transfection with GFP labelled miR-449a (d3, 4) mimic greatly reduces the number of GFAP and DCX positive cells in 3% FBS enriched medium (d4), compared to d2. The DCX labelled differentiated mBTSC (d4 inset) was spared from (GFP labelled) miR-449a mimic. In keeping, knockdown of Gpr158 abolishes GFAP and DCX expression on mBTSC (c6). **e** quantification of DCX (red bars) and GFAP (green bars) positive cells in miR-449a antagomir or GPR158 overexpressing *Pten/p53* mBTSC. E, quantification of DCX (red bars) and GFAP (green bars) positive cells in miR-449a mimic or GPR158 knock-down *Rb/p53* mBTSC

Under growth-promoting conditions in EGF, FGF enriched serum-free medium, miR-449a suppresses Ccnd1 and inhibits migration and invasion, suggestive of a tumour-suppressive effect. This effect is seen in cell lines derived from somatic cancers, such as non-small lung cancer [18], hepatocellular carcinoma [16], colon cancer [27] or neuroblastoma [19]. A different role of miR-449a emerges from studies on CNS development where high miR-449a levels are associated with neural progenitor expansion and suppression of neural differentiation [25, 26], possibly through an inhibition of GPR158. Therefore, we first determined Gpr158 and Ccnd1 expression in *Pten/p53* or *Rb/p53* mBTSC, under proliferative (serum-free, EGF, FGF enriched) and differentiating (3% FBS) conditions. Immunofluorescent labelling for Gpr158 shows virtually no labelling under proliferative conditions (Fig. 5a) and upregulation when grown in differentiating conditions, whilst density of nuclear labelling for Ccnd1 markedly decreased upon culturing under differentiation conditions. Quantification confirms upregulation of *Gpr158* and downregulation of miR-449a upon induction of neural differentiation (Fig. 5a). Next we investigated the effect of miR-449a on neural differentiation on individual cellular level, again comparing serum-free, EGF and FGF-enriched growth-promoting environment with differentiation-inducing culture conditions containing foetal bovine serum (FBS) (Fig. 5c). Gpr158 overexpression in *Pten/p53* or *Rb/p53* mBTSC induces neural differentiation, demonstrated by the detection of doublecortin (DCX), a protein expressed in migrating neuroblasts and immature neurons [41]. Immunolabelling for DCX in lentivirus-*Gpr158* transduced *Rb/p53* mBTSC confirms expression (Fig. 5b) and qPCR profiler expression analysis of these cells shows upregulation of genes associated with proneural signature *Rb/p53* mBTSC (miR-449a^high^). Dual immunofluorescence of *Pten/p53* or *Rb/p53* mBTSC, for GFAP and DCX shows virtually no positive cells in EGF, FGF-enriched serum-free medium even with miR-449a antagomir transfection or GPR158 transduction (Fig. 5c,d). Induction of the cells with 3% FBS marginally increases GFAP and DCX positive cells in *Rb/p53* mBTSC and much stronger in *Pten/p53* cells, consistent with the different baseline levels of miR-449a in the two mBTSC lines (Figs. 5c2, d2, E, F). However, *Rb/p53* (miR-449a^high^) mBTSC transfected with miR-449a antagomir rescues the suppression of neural differentiation, and in keeping, *Pten/p53* (miR-449a^low^) mBTSC transfected with miR-449a mimic (Fig. 5d4) showed a marked reduction compared to empty vector-transfected controls (Fig. 5d2). Cells spared from transfection with miR-449a mimic (Fig. 5d4) conspicuously retained their expression of DCX, suggesting a direct effect of miR-449a to suppress neural differentiation. The same effect is seen when Gpr158 expression was inhibited with *Gpr158* siRNA. Furthermore, we confirm these effects when cells were subjected to induction of neuronal (retinoic acid and forskolin) and glial differentiation (LIF/BMP2) (Supplementary Figure 2). Here, glial and neuronal differentiation was seen after suppression of miR-449a expression with antagomir (Supplementary Figure 2F, G, O) or overexpression of Gpr158 (Supplementary Figure 2H, J, P, R). An inverse experimental setup was chosen for *Pten/p53* mBTSC (miR-449a^low^) (Supplementary Figure 2B-D, and K-M), confirmed by cell quantification (Supplementary Figure 2I, J, Q, R).

In conclusion, the neurogenic effect of Gpr158 can be antagonised by mir-449a. This effect is modulated by growth conditions, whereby a proliferation-inducing environment with EGF, FGF-enriched serum-free medium is not permissible neurogenic effects of GPR158. Under these conditions, downregulation of Ccnd1 explains the miR-449a-induced reduction of growth and migration. Instead, in an environment permissive for neural differentiation, miR-449a and Gpr158 are antagonists where miR-449a inhibits, and Gpr158 promotes neural phenotypes.

### miR-449a downregulates Gpr158 and Ccnd1 in allografts in vivo

**Fig. 6:**
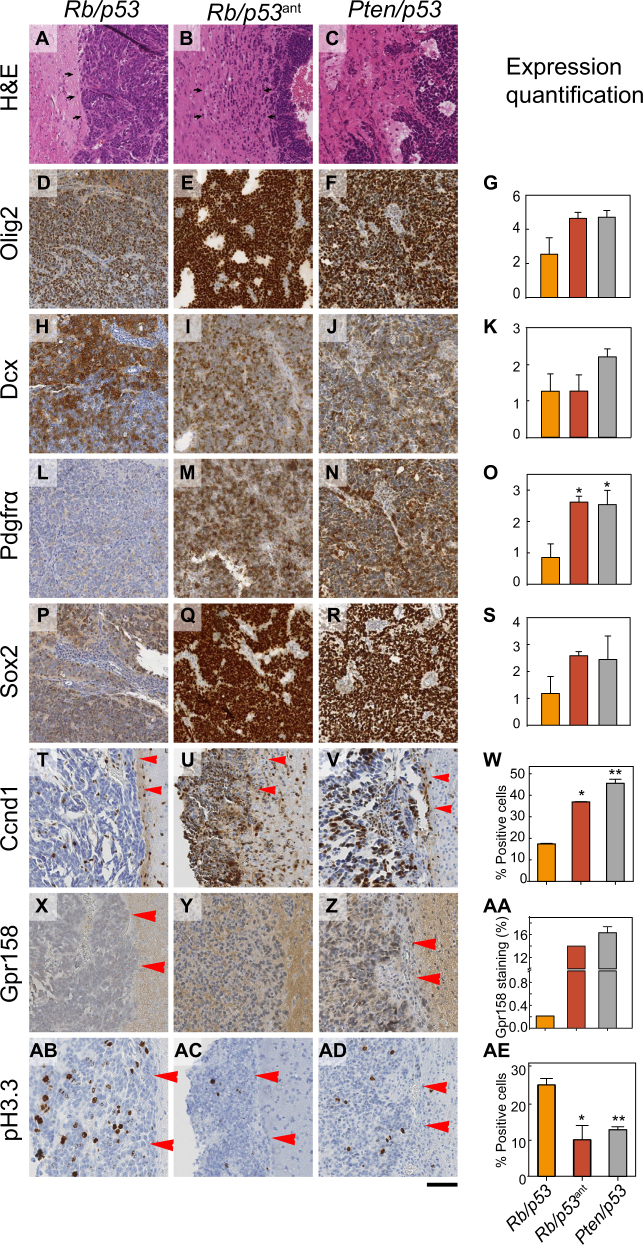
Expression of neural markers, Gpr158 and Ccnd1 in allografts of miR-449a^high^ and miR-449a^low^ tumours. Phenotype of tumours generated from allografted *Rb/p53* (left column), miR-449a antagomir-treated *Rb/p53*^ant^ (centre column) and *Pten/p53* cells (right column) into NOD-SCID mice. **a** Morphology (H&E) shows a well demarcated tumour border in *Rb/p53* PNET (single line of arrowheads), and an infiltrative margin in both, antagomir-treated *Rb/p53* grafts (**b**) and *Pten/p53* grafts (**c**). Image analysis of regions of interest on whole slide digitized images of immunostained histological sections with Definiens Developer shows a reversal of *Rb/p53*^ant^ grafts to levels found in *Pten/p53* grafts (markers Olig2 (**d–g**), Pdgfrα (**l–o**), and Sox2 (**p-s**), whilst only DCX it is unaltered (**h–K**). Expression of these markers is reduced in *Rb/p53*; miR-449a^high^ tumours, high in *Pten/p53* (miR-449a^low^) grafts, and restored upon suppression of miR-449a in antagomir-treated *Rb/p53*^*ant*^; miR-449a^low^ grafts. (**t-ae**) miR-449a regulates Ccnd1 and GPR158 in tumours in vivo: (**t–w**), Ccnd1 expression is low in *Rb/p53* (miR-449a^high^) tumours (**t**), and high in *Pten/p53* (miR-449a^low^) tumours (**u**) as well in antagomir-treated *Rb/p53*; miR-449a^low^ tumours (**v**, **w**). Gpr158 expression is regulated correspondingly (**x**–**aa)**. In keeping with the shorter survival of mice with *Rb/p53*; miR-449a^high^ tumours, proliferation is high in these tumours (**ab**). Scale bar 50 µm. All figures: *p ≤ 0.05; **p ≤ 0.01; ***p ≤ 0.001 (Student’s *t*-test). Each value represents the mean ± s.d

We further confirmed the interaction of miR-449a with Ccnd1 and Gpr158 in vivo. Allografts of *Rb/p53* (miR-449a^high^), *Pten/p53* (miR-449a^low^), and *Rb/p53*^*ant*^ (miR-449a^low^) were generated in NOD-SCID immunodeficient mice. *Pten/p53* allografts have a (pro)-neural phenotype and diffusely infiltrate the CNS. *Rb/p53* allografts are poorly differentiated, lacking distinctive glial and neural marker expression and grow sharply demarcated against the CNS (Fig. 6a, arrowheads). *Rb/p53*^ant^ grafts reverted to an infiltrative phenotype (Fig. 6b arrowheads), similar to *Pten/p53* allografts (Fig. 6c). Quantification of expression on immunolabelled tissue sections with whole slide imaging and image analysis [6] corroborates the RNA expression data of *Pten/p53* and *Rb/p53*^ant^ tumours with their high expression of Olig2, PDGFRα and Sox2, whilst *Rb/p53* grafts expressed much lower levels of these markers with quantification data in the right column (Figs. 6g, k, o, s). miR-449a targets CCND1 and Gpr158 are highly expressed in tumours with low miR-449a levels (i.e., in *Pten/p53* and *Rb/p53*^ant^) whilst it is much less expressed in *Rb/p53* (miR-449a^high^) tumours, consistent with the in vitro experiments (Fig. 5). In conclusion, miR-449a directly downregulates Gpr158 and Ccnd1 in vivo (Figs. 6t, x), reducing neural phenotypes.

### GPR158 is highly expressed in the human CNS, and differentially expressed in human oligodendrogliomas, astrocytomas and glioblastomas

**Fig. 7:**
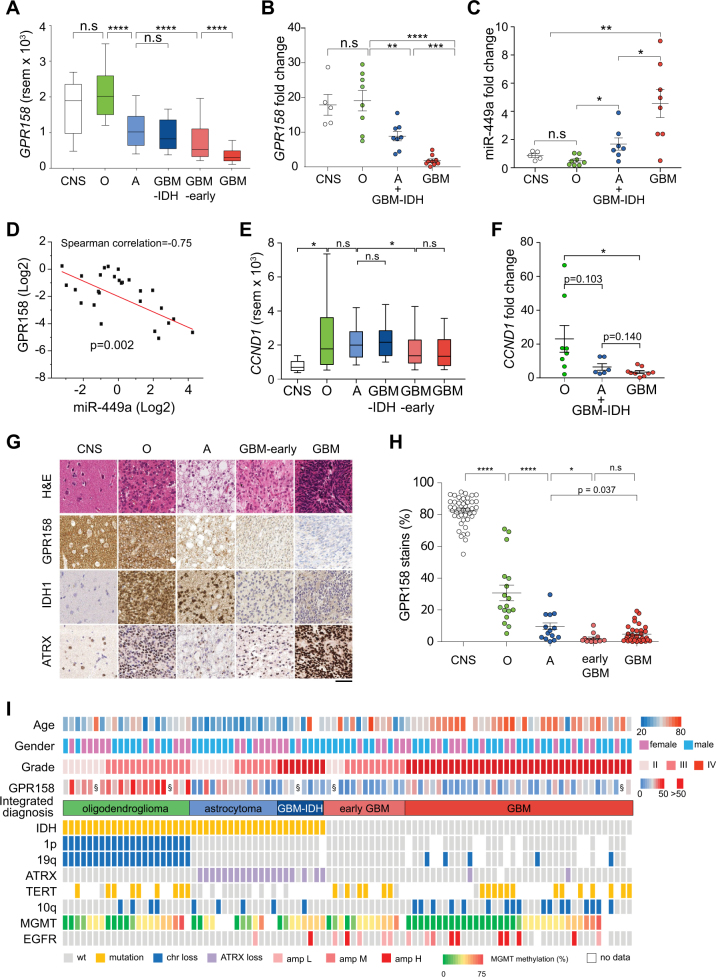
Analysis of GPR158 expression in tumour and control brain samples. Expression of GPR158 in CNS, and the five glioma subgroups oligodendroglioma, astrocytoma, GBM-IDH, early GBM and GBM, as defined by the integrated (morphological and molecular) diagnosis. **a** TCGA RNA sequencing (rsem) data indicate highest GPR158 expression in CNS (*n* = 5) and oligodendrogliomas (O, *n* = 84), a reduction in astrocytomas (A, *n* = 138), GBM-IDH (*n* = 9), and further down-regulation in 'early GBMs' (*n* = 54) and GBMs (*n* = 148). The differences between O, A/GBM-IDH, early-GBM and GBM (both IDH^wt^) are highly significant. (**b**) GPR158 RNA expression in frozen CNS and glioma tissue from our institution. Relative GPR158 expression levels measured by RT-qPCR is consistent with TCGA rsem data. **c** Expression levels of miR-449a in the same samples, and (**d**) plot of inverse correlation of miR-449a and GPR158 RNA expression levels. **e** CCND1 expression is higher in gliomas than in CNS but not significantly differentially expressed across all glioma groups in TGCA samples. **f** in our samples, CCND1 expression is lower in GBM than in oligodendrogliomas, in keeping with the observation in our brain tumour allografts (Figs. 6t, u, v) that the proliferative Rb/p53 tumours downregulate Ccdn1. **g** Representative histology and immunostaining patterns in tumours (*n* = 93) from our institution. GPR158 immunoreactivity is strong in CNS and oligodendroglioma, much weaker in astrocytoma, and nearly negative in IDH wild-type 'early' GBM and GBM. Mutant IDH1 is expressed in oligodendrogliomas and astrocytomas, but not in CNS, and glioblastomas. ATRX is lost only in IDH mutant astrocytomas. All other tumours and the CNS maintain ATRX expression. Scale bar corresponds to 100 µm. **h** Quantification of protein expression by whole slide imaging and image analysis of tissue sections immunostained for GPR158. CNS tissue shows the highest expression, followed by oligodendrogliomas and astrocytomas, whilst there is a significantly lower expression in IDH wild-type early-GBM and GBM, consistent with the RNA expression data shown in (**a** and **b**). Oligodendroglioma (*n* = 17), astrocytoma (*n* = 16), early-GBM (*n* = 12) and GBM (*n* = 34). CNS tissue data were obtained from tissue fragments within some of the resection specimens containing normal CNS. **i** Overview and summary of demographic parameters, tumour grade, integrated diagnosis and molecular profile of the tumours analysed in (**e**). There are two types of IDH mutant gliomas, oligodendrogliomas, defined by a loss of chromosomal arms 1p and 19q (1p/19q codeleted) and typically with a mutation in the telomerase reverse transcriptase (TERT) promoter, and astrocytomas or glioblastomas (GBM) which carry a mutation of alpha thalassemia/mental retardation syndrome X-linked (ATRX) resulting in functional loss of the protein. Patients with IDH mutant tumours are younger than those with IDH wild-type GBM. GPR158 levels are highest in oligodendrogliomas, lower in astrocytomas and lowest in GBM, as described above

To establish the roles of miR-449a and GPR158 in human gliomas, we first analysed 'RNA-Seq by Expectation-Maximization' (rsem) data of 431 gliomas (155 GBM and 276 low-grade gliomas (LGG), Supplementary table 3) from the TCGA database, and complemented this dataset with mRNA expression analysis and immunohistochemical detection of GPR158 protein in human gliomas from our institution (NHNN, Supplementary table 3). To correlate GPR158 expression with clinically, diagnostically and biologically relevant tumour entities [2], we defined oligodendrogliomas (O, *n* = 83) as IDH mutant, 1p/19q co-deleted tumours, and astrocytomas (A, *n* = 138) as IDH mutant, ATRX mutant tumours with no 1p/19q codeletion. As the WHO grade is of disputed clinical significance [42, 43] in astrocytomas, we did not further stratify these tumours by WHO grades II or III. IDH mutant GBM (GBM-IDH, *n* = 9) which carry a poorer prognosis than IDH mutant astrocytomas, were grouped separately from astrocytomas. IDH wild-type high grade gliomas with 7p gain, 10q loss, EGFR amplification and TERT promoter mutation were considered as GBM (corresponding to WHO grade IV) (*n* = 146). Histological lower grade IDH wild type astrocytomas with molecular profiles of glioblastoma are considered as 'early stage' GBM [44] (eGBM; *n* = 54). Using these criteria and TCGA RNA sequencing data, we investigated GPR158 expression in these glioma subtypes. GPR158 expression was highest in the CNS (*n* = 5) and in oligodendrogliomas, followed by astrocytomas (*p* < 0.0001), and significantly lower in eGBM and GBM (*p* < 0.0001; Fig. 7a, Supplementary Figure 3A-C). The difference of *GPR158* expression levels was statistically significant between eGBM and GBM (*p* < 0.0001), suggesting that *GPR158* was further down-regulated upon tumour progression to a higher (histological) grade. In 29 other TCGA tumour entities (Supplementary Figure 3D), with the exception of pheochromocytoma and paraganglioma [45], the remaining tumours types expressed very little or no *GPR158*. In conclusion, we show here that there is a statistically significant difference of *GPR158* expression between clinically and biologically distinct glioma subgroups, and *GPR158* expression was specific in nervous system-related tumours. mRNA expression data of miR-449a and GPR158 were confirmed on 25 frozen glioma samples and 5 CNS samples from our own collection (miR-449a: CNS, *n* = 5; O, *n* = 7; A, *n* = 7; GBM, *n* = 8; and GPR158 CNS, *n* = 5; O, *n* = 8; A/GBM-IDH, *n* = 8; GBM, *n* = 9) (Figs. 7b, c) by RT-qPCR analysis. miR-449a inversely correlates with GPR158 expression (Figs. 7b, c, d), consistent with previous experiments. To assess, if the expression of the miR-449a target CCND1 may also correlate with tumour grade and type, we retrieved expression data from TCGA. CCND1 rsem is low in CNS tissue, and slightly increased within a wide range of expression levels across all subgroups of gliomas, with no statistically significant difference between oligodendrogliomas, astrocytomas and IDH wild-type glioblastoma (Figs. 7e, f). Thus, the expression of CCND1 remains largely independent of the tumour subtypes, supporting the notion that GPR158 may have a role as biomarker that is independent from the miR-449a target *CCND1*.

To confirm GPR158 protein expression on paraffin-embedded tissue sections by immunohistochemical (IHC) detection, we tested 85 samples from our archive, all molecularly characterized (IDH1/2, Histone H.3.3, BRAF, TERT promoter and 1p/19q, 7p/EGFR, 10q copy numbers, ATRX IHC, Fig. 7g). Again, GPR158 expression strongly correlated with histological entities (Figs. 7g, h, i) being highest in CNS cortex, followed by oligodendrogliomas, whilst astrocytomas stained much weaker, but had still a clearly visible fine granular cytoplasmic stain. eGBM and GBM showed no staining or only focal, patchy weak and diffuse staining (no statistically significant difference between both groups). Small fragments of CNS cortex, present in many samples, in particular of surgical aspirate, served as internal technical control and were used to generate quantitative CNS grey matter expression data by whole slide image analysis (Fig. 7h) [6], and also correlate with TCGA and our own expression data (Figs. 6a, b, c). In conclusion, we show here that GPR158 RNA and protein expression correlate with distinct diagnostic glioma entities, suggesting that GPR could represent a biomarker for stratifying these tumours (Fig. 7i).

### Lower miR-449a and higher GPR158 expression correlates with longer survival of glioma patients

**Fig. 8:**
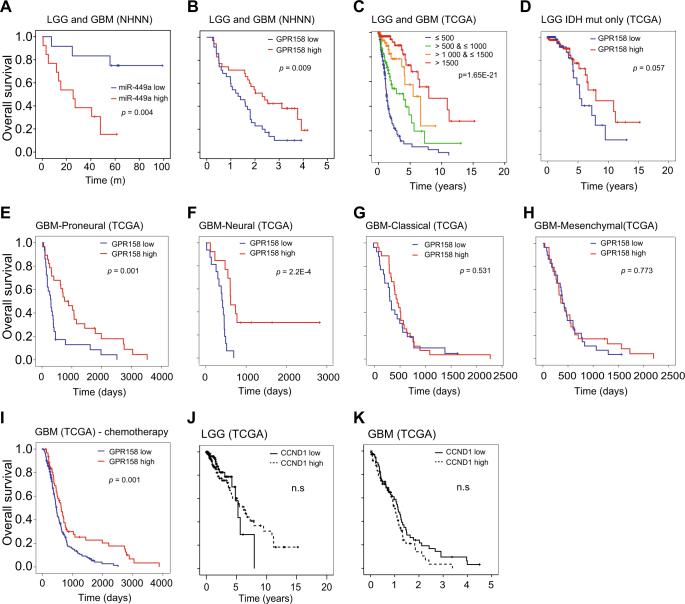
Patients with gliomas expressing higher level of GPR158 survive longer and show better response to chemotherapy. **a** Kaplan–Meier estimates of overall survival in all grades of gliomas from our institution, grouped by miR-449a expression levels or by (**b**) GPR158 expression measured by staining intensity. Patients with tumours expressing low levels of miR-449a or high levels of GPR158 survive significantly longer. The 'miR-449a low' and 'miR-449a high' groups, and the 'GPR158 low' and 'GPR158 high' groups were separated by the median of expression levels or staining intensity. **c** Stratification of survival according to GPR158 expression levels (rsem) in the TCGA cohort. The survival pattern from TCGA data confirms the finding in (**a**). **d** The cohort of patients (from TCGA) with IDH mutant tumours expressing high levels of GPR158 show a trend of longer survival than low expressors. However, in this cohort the difference does not reach significance level. **e** Proneural and **f** neural subtypes of GBMs (from TCGA) with higher expression of GPR158 show a significantly longer survival, whilst no difference is seen in GBM of (**g**) classical (**h**) or mesenchymal profiles. **i** GBM with high GPR158 expression show a significantly better response to chemotherapy. All *p* values were estimated using log-rank test. **j** Low grade or **k** high grade gliomas, stratified by CCND1 expression do not show a difference in survival, indicating a role mediated by the miR-449a target GPR.158 rather than CCND1

Finally we evaluated if miR-449a and GPR158 also have a role as potential prognostic biomarker, i.e. correlate with patient survival (Fig. 8). First we separated the 25 NHNN patients of whom we had miR-449a expression values into 2 groups (above and below median) and found significantly better survival in the miR-449a^low^ group (*p* = 0.004) (Fig. 8a). Next, we separated the 85 NHNN patients of whom we had tissue stainings into two groups according to the GPR158 stain score (above and below median), and found that patients whose tumours expressed higher levels of GPR158 survived longer (*p* < 0.05; Fig. 8b). Then we accessed the TCGA repository, retrieved and quantified *GPR158* mRNA expression levels as per rsem (Supplementary table 3), and assigned patients to 4 strata according to their *GPR158* rsem level (≤500 (Interval 1), >500 ≤ 1000 (Interval 2), >1000 ≤ 1500 (Interval 3) and >1500 (Interval 4)). There was a strong correlation between *GPR158* expression level and survival across all tumours (*p* = 1.65E-21; Fig. 8c). Patients with high GPR158 expression were younger than those with low expression, consistent with IDH mutant tumours occurring in younger patients are also being associated with better survival. There is no significant association of *GPR158* expression with gender (Fisher’s exact test, *p* = 0.158-0.359, one interval vs. all other three intervals, Supplementary table 3). To identify if GPR158 has a prognostic role within either IDH wild-type or IDH mutant subgroups, we then grouped patients according to the IDH mutation status. Although not reaching significance in the IDH mutant cohort, GPR158^high^ tumours showed an obvious tendency to longer survival (*p* = 0.057; Fig. 8d). However, in the IDH^wt^ eGBM subgroup, GPR158^high^ tumours showed no different survival from GPR158^low^ tumours, as only few patients were available in the GPR158^high^ group. We assessed the prognostic value of GPR158 in the four subtypes of GBM [4] and found significantly longer survival of GPR158^high^ patients in the proneural and neural groups (Fig. 8e and Supplementary table 4), but not in the other two groups (Figs. 8g, h), and there is no influence of *CCND1* expression on survival (Figs. 8j, k). In summary, in both the NHNN and the TCGA glioma cohorts, we confirmed that higher GPR158 transcript and protein expression levels correlate with better survival, and patients with GPR158^high^ IDH^wt^ GBM responded significantly better to chemotherapy compared to patients with GPR158^low^ tumours (Fig. 6i, Supplementary table 4).

## Discussion

**Fig. 9:**
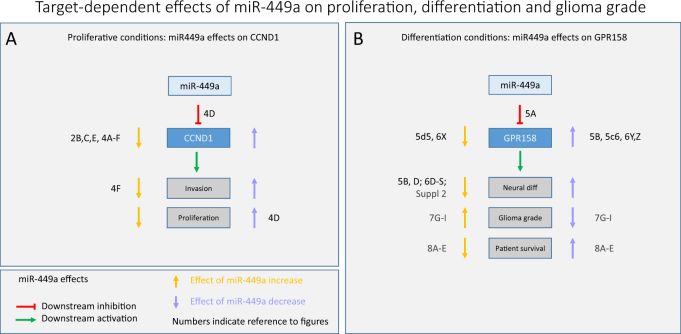
Target dependent effects of miR-449a on proliferation, differentiation and glioma biology (**a**) in stemness—maintaining growth conditions with EGF, FGF enriched serum-free medium, miR-449a—mediated effects are predominantly exerted through CCND1 inhibition, thus reducing invasion and proliferation. For example, miR-449a^high^ *Rb/p53* cells have low expression levels of Ccdn1, proliferate and migrate slower, and have less self-renewal capacity. **b** in growth conditions promoting neural phenotypes in vitro, such as FBS, Forskolin, retinoic acid and in vivo experimental settings and humans gliomas, miR-449a suppresses GPR158, reducing neural marker expression, and is associated with higher glioma grade and shorter survival. Numbers and letters provide a reference to figures in the text

To identify differentially regulated genes and their targets in gliomas, we used mouse models which develop two well-defined tumour phenotypes—gliomas *(Pten/p53)* or PNET *(Rb/p53)*—with distinctive lineage, growth rate and invasiveness [6, 7]. In a microRNA screen, we identified miR-449a as significantly differentially regulated. miR-449a belongs to the miR-34/449 family, and shares with miRNA-34, 449b, and 449c seed sequences, secondary structures [46], and downstream targets, including *CCND1* and E2F transcription factor 5 (*E2F5*) [27, 47–49]. To understand the regulatory function of miR-449a in BTSC, we looked for targets relevant for intrinsic brain tumours (Fig. 2). We confirmed and validated the known target *CCND1* [20] (encoding Cyclin D1) (Figs. 2e and 4a–f) and we identified a new target, G-protein coupled receptor 158 (*GPR158*) which is downregulated by miR-449a (Figs. 2c, e, f). GPR158 was considered highly relevant, as it is most closely related to GABA receptors [28], is widely and strongly expressed in the central nervous system and has a role in cognition [50]. It is highly expressed in the CNS and low in somatic organs https://www.proteinatlas.org/search/GPR158, and it is also highly expressed in 'low grade glioma' and in phaeochromocytoma/paraganglioma, a neuroendocrine neoplasm. Instead it is reduced in glioblastoma and expressed at even lower levels in a malignant childhood tumour, AT/RT (Supplementary Figure 3D, E). We show here that miR-449a has target-dependent effects on cell migration, proliferation and differentiation, mediated by CCND1 or GPR158 (Fig. 9). miR-449a inhibits CCND1 under proliferative conditions in stem cell medium (serum free, EGF, FGF supplemented), reducing proliferation and migration (Figs. 3f, 4a–f and 5a). It also inhibits GPR158, suppressing neural differentiation (Fig. 3f). Under these conditions, however, GPR158 overexpression reverses miR-449a—induced phenotypes (caused by CCND1 inhibition) by promoting neural differentiation and apoptosis, thus further reducing migration and proliferation (Figs. 4g-s) and this creates a seemingly paradox result. This paradox has been addressed by further studying the effects of miR-449a and GPR158 in conditions facilitating neural differentiation in vitro and in vivo. Under these conditions, GPR158 induces neural, glial and neuronal phenotypes and apoptosis (Figs. 4k-s and 5, Supplementary Figure 2) in tumour spheres and cells and in allografts in vivo (Fig. 6). This can be antagonised by miR-449 mimics (Fig. 5, Supplementary Figure 2). GPR158 knockdown has opposite effects, reducing neural differentiation, apoptosis and increasing growth (Figs. 4k–o, and Supplementary Figure 2), whilst miR-449a antagomir restores a neural phenotype (Fig. 5c4, Supplementary Figure 2). This antagonistic effect between mir-449a and GPR158 was consistently found in conditions promoting neural (FBS, Fig. 5), glial (LIF/BMP2), and neuronal (retinoic acid/forskolin) phenotypes (Supplementary Figure 2) in vitro, and in murine allografts in vivo (Fig. 6).

In conclusion, the effects of miR-449a are modulated by growth conditions and environment of BTSC (Fig. 9). Our data raise the possibility that the effects of miR-449a may be target-dependent, acting on CCND1 or GPR158 (Fig. 9). miR-449a has an inhibitory effect on migration and invasion in vitro in some cancer types [19, 20, 23] and Figs. 3d, e). In vivo, miR-449a has a tumour suppressive effect in some cancers, such as hepatocellular carcinoma, [16], or lung cancer [18], but not in others, where an association with cancer progression was found, such as breast [51] or colorectal cancer [52]. A previous study on brain tumours shows results similar to our in vitro data [17], whilst a discrepancy of miR-449a correlation with brain tumour grade is noted. In that study tumour types were not specified and thus the results may not be directly comparable to our cohort which used molecularly characterized and stratified tumours. A yet different role of miR-449a has been identified in the developing neuroepithelium, where a transient upregulation is associated with neural progenitor expansion in the rat brain at embryonic day 10 [53] and suppression of neural cell fate activates choroid plexus.

CCND1 can have context-depending roles in vitro and in vivo: It is one of the major regulators of the cell-cycle progression, can act as an oncogene, and aberrant expression is commonly seen in human cancers [54, 55]. In keeping, in vitro under appropriate conditions CCND1 promotes proliferation and migration of BTSC (Figs. 4a–c, 5a and 9a), and is modulated by miR-449a expression, i.e., is higher in *Pten/p53* mBTSC (miR-449a^low^) than in *Rb/p53* mBTSC (miR-449a^high^) (Figs. 4c and 5a) and in miR-449a^high^ experimental tumours, Ccnd1 is downregulated (Fig. 6t). Instead, in neural differentiation conditions Ccnd1 is downregulated (Fig. 5a) in keeping with overall reduction of cell proliferation and increased neurogenesis [56, 57] and differentiation into astrocytes [58]. This is consistent with our observation of higher expression of neural markers, and of GPR158 and CCND1 in miR-449a^low^ tumours developing from allografted *Pten/p53* cells and from *Rb/p53* cells treated with miR-449a antagomir (Fig. 6). However, unlike in many other cancers, CCND1 is not differentially expressed in different grades and types of brain tumours (Fig. 7e), and TCGA outcome data do not show a difference in survival between patients with gliomas expressing low or high CCND1 levels (Figs. 8j, k).

An important finding of our study is the inverse correlation of miR-449a and GPR158 expression (*p* = 0.002, Fig. 7d). Of translational importance, miR-449a expression correlates with tumour grade (Fig. 7c) and with poorer survival (*p* = 0.004, Fig. 8a), and consistent with our experimental data, GPR158 expression correlates with better prognosis (Figs. 8b-i) and inversely with tumour grade (Figs. 7e, i, h and 9b). Our results are discrepant to those of a study on prostate cancer where increased expression of GPR158 correlates with poorer survival [31]. This could be related to the specific pathobiology of prostate cancer, where enrichment of GPR158 expressing neuroendocrine cells (thought to represent transdifferentiated prostate epithelial cells) show more aggressive clinical behaviour and thus are associated with poorer survival. In contrast, expression of GPR158 and (pro-) neural markers is high in low-grade gliomas and typically much lower in GBM. Correlation of GPR158 expression levels to GBM, stratified according to the molecular subtypes [4] further supports this notion, as GPR158 expression is highest in the proneural subtypes (a class that is enriched for IDH mutations and PDGFR amplifications), and decreases in classical (enriched for *EGFR* amplified and *CDKN2A* mutant tumours) and mesenchymal subtypes, which are most commonly *NF1* mutated (Supplementary Figure 3c). The neural subtype surprisingly appears close to the classical and mesenchymal type, and this could be ascribed to a possible “contamination” of the individual subclasses [59], or superimposed effects of the tumour microenvironment [60]. In keeping with these observations, the expression analysis with the mouse Profiler PCR Array showed a significant up-regulation of *Map2*, *Sox2*, and *Pdgfra* (Fig. 4q) in Gpr158 expressing cells, and corresponding protein expression in tumours developing from allografted cells in vivo (Fig. 6), thus further strengthening the correlation of our experimental data with clinical outcome data.

A further difference between prostate cancer and brain tumours is that in the former, the C-terminal portion of GPR158 is translocated to the nucleus whilst we did not observe nuclear staining in CNS grey matter or in glioma cells using a specific anti-GPR158 C-terminal antibody. This discrepancy could be explained by different signalling pathways relevant in prostate and brain tissues. A role of GPR158 long non-coding antisense (AS) RNA in lung cancer has recently been described, where high expression of GPR158 AS1 correlates with poorer overall survival [32]. In contrast, we show that GPR158 AS1 expression level positively correlates with GPR158 mRNA level (Supplementary Figure 3F). It has been shown previously that antisense RNA can act both, enhancing and suppressing and this is possibly related to differences in tissue specificity [61, 62].

In conclusion, we identified through a phenotypic screen the highly differentially regulated miR-449a, which targets CCND1 and GPR158 and has target-dependent functions (Fig. 9). It is highly expressed in a growth-promoting environment in vitro, reducing proliferation and invasion. In a neurogenic environment in vitro and in tumours in vivo it inhibits neural phenotypes. We show that miR-449 directly targets and downregulates CCND1, resulting in reduced proliferation in vitro, and GPR158, antagonising neural differentiation and apoptosis in glioma stem cells. High miR-449a expression levels correlate with shorter survival, whilst high GPR158 expression is associated experimentally with neural phenotypes, cell differentiation and clinically with lower glioma grades and better patient survival and may serve as predictive biomarker. miR-449a could be considered as druggable target, e.g., using antagomirs, and GPR158, a member of a large family of receptors may be targeted by pharmacological agents [63, 64], e.g., by stimulating the downstream pathway of GPR158, to reduce tumour growth.

## Materials and methods

### Animals

All procedures performed on mice were according to Institutional and UK Home Office guidelines (Project licenses 70-7428 and PA79953C0). The ARRIVE guidelines were followed as part of the institutional policy and the licensing of the experiments. Mice carrying *p53*^*loxP/loxP*^, *Rb*^*loxP/loxP*^ [65] or *Pten*^*loxP/loxP*^ [66] transgenes were intercrossed resulting in co-deletion of *Rb/p53* or *Pten/p53* and the *ROSA26-lacZ*^*loxP/loxP*^ reporter gene upon cre-mediated recombination. Tumours were induced and BTSC derived as described [7].

### Microarray preparation, hybridization and data analysis

Total RNA used for microarray was extracted from frozen tumours using TRIzol [6]. miRNAs from 600ng of total RNA were labelled with Hy3 or Hy5 fluorophores according to manufacturer’s protocol (miRCURY LNA microRNA Hi-Power Labelling kit, Exiqon). Pair-wise RNA samples labelled with Hy3 or Hy5 dye were hybridized to the miRCURY LNA microRNA Array 7 (Exiqon). Spike-ins were used for array quality control. Microarrays slides were scanned (G2565BA Agilent) and images quantified using ImaGene 9 (Exiqon, Denmark). Background signals were corrected using *normexp* method as described before [67] with limma package on R. Expression validation by quantitative real-time-PCR on a LightCycler 480 (Roche). Amplification curves were analysed using the Roche LC software and normalized using five normalizers (Supplementary table 1) to determine the −ΔΔCp value.

### Validation of miRNA targets by Ago2 and biotin double pull-down assay and luciferase assay

Direct miRNA-mRNA interaction was confirmed by double pull-down assay [35]. 8 × 10^6^ mBTSC were transfected with 450 pmol of biotin-labelled miR-449a mimic (Exiqon). 10% of the lysate was used as input RNA (Fraction 1), 90% for double pull-down. The first pull-down was on Argonaute TISC-Catalytic Component 2 (Ago2) immunoprecipitation with Protein G Dynabeads (75 μl; Invitrogen). For the second pull-down, Dynabeads were incubated with Relay Samples 2 and 3. RNA was eluted (miRCURY RNA Isolation kit, Exiqon) to obtain Fractions 2 and 3. Ccnd1 and Gpr158 was quantified by qRT-PCR. For the luciferase assay, the 3′ UTR of Gpr158 (174 bp) containing wild-type or mutant miR-449a-5p binding site were synthesized commercially (GeneArt, Invitrogen). The 3′ UTR fragment was inserted to pMIR-REPORT luciferase vector and reporter assay was carried out 48 h post-transfection (Dual-Light system, Applied Biosystems).

### Cell proliferation, gap closure assay and migration assays

Assays were performed on IncuCyte (Essen bioscience, US) in 96 well plates. Proliferation: 1500 cells per well; Gap closure assay: 75,000 cells per well, gap generation 24 h post-seeding (wound maker, Essen bioscience) and mitomycin C (0.01 mg/ml) was added. Chemotactic migration assay: 3000 cells seeded on a ClearView chemotaxis plate (Essen bioscience). The bottom was filled with attractant medium (40 ng/ml growth factor). Plates were scanned and analysed using Incucyte Chemotaxis module. Collagen-based invasion assay: 5000 cells per well in a U-bottom 96-well plate in 100 μl of 20% (v/v) methylcellulose in culture medium to form a sphere (24 h). Three hours after embedding on fibrillary bovine collagen (2.1 mg/ml), the spheres were repeatedly imaged and quantified with Image J.

### Knock-down or overexpression of miR-449a, CCND1 and GPR158

Murine brain tumour stem-like cells (mBTSCs) were transfected with (i) LNA-miR-449a antagomir/inhibitors (Exiqon), (ii) LNA-miR-449a mimics (Exiqon), (iii) mouse Gpr158 siRNA (Life Technology Co.), (iv) CCND1 siRNA (Cell Signaling), or (v) scramble controls using Viromer Black (lipocalyx) according to the manufacturer’s protocol. After 24 h, cells were used for proliferation, gap closure, invasion, tumour-sphere, or differentiation assay; alternatively total RNA was extracted for transcript quantification after 48 h.

The lentiviral vector containing short hairpin RNA (shRNA) for stable inhibition of human GPR158 and the control vector were purchased from the UCL RNAi library. Mouse Gpr158 cDNA clone in entry vector pENTR223.1 was purchased from DNASU, and cloned into pLX301 lentiviral vector. Lentivirus was produced in HEK293T cells using Fugene according manufacturer’s protocol (Promega). The human GPR158 expression vector was a kind gift from Elizabeth Fini, University of Southern California, Los Angeles [30].

### Human tissue resources

The use of human tissue samples was licensed by the NRES, University College London Hospitals NRES license for using human tissue samples: Project ref 08/0077 (S.B.). The storage of human tissue is licensed by the Human Tissue Authority, UK, License #12054 to SB. hGBM-IC were derived and cultured as described [68]. Glioma tissue blocks and associated clinical and molecular information were from the archives of the NHNN.

### Extreme limiting dilution assay

Cells were plated in 96-well plates at 1, 2, 5, 10, 20, 50, 100, and 200 cells/well as described previously [69], and cultured in 100 µl of serum-free medium per well as mentioned above. The percentage of wells with neurosphere formation was determined 7 days post seeding. Stem cell frequency was estimated using software available at http://bioinf.wehi.edu.au/software/elda/ [39]. Further instructive information can be found on this informal resource http://www.biology-pages.info/L/LimitingDilution.html

### Differentiation assay of cells overexpressing or knockdown for GPR158 or for miR-449

Murine brain tumour stem cells of the *Rb/p53* or *Pten/p53* genotypes transduced with lentivirus expressing GPR158 or GFP as control, containing puromycin a selection marker (4 weeks selection). 24 h after transfection/transduction growth medium was exchanged with differentiation medium containing DMEM/F12, 2% B27, 1% penicillin-streptomycin, and supplemented with 3% FBS, or 10 uM RA (Sigma, R2625) and 20 uM Forskolin (Abcam, ab120058) for neuronal differentiation, or with 50 ng/ml LIF (Santa Cruz, sc-4989) and 50 ng/ml BMP2 (Thermo Fisher, PHC7145) for astrocytic differentiation [70]. After 48 h differentiation cells were fixed and stained for doublecortin (DCX, ab18723, 1:800, Abcam) and GFAP (ab4674, 1:1000, Abcam), followed by secondary antibodies conjugated with Alexa dyes.

### RT-qPCR and mouse neurogenesis profiler array

cDNA was synthesized using RevertAid RT kit (Thermo Fisher Scientific Inc.). RT-qPCR was performed in triplicate with SYBR Green Mastermix (Thermo Fisher Scientific Inc.). The primers are listed in Supplementary table 5. Gene expression was normalized against GAPDH levels and fold changes were calculated using the 2^-ΔΔCt^ method on DataAssist 3.1 software (Thermo Fisher).

### Caspase-3/7 activity assay

BTSCs or GBM primary cells with different expression levels of Gpr158 (or GPR158) were lysed using lysis buffer (#7018; Cell Signaling). Subsequently, 40 μg of the samples was diluted to a final volume of 150 μl with protease assay buffer (20 mM HEPES (pH 7.5), 10% glycerol, and 2 mM DTT), and supplemented with 20 μM caspase-3 preferred, fluorogenic substrate Ac-DEVD-AMC (#556449, BD Pharmingen) for a 2-hour incubation 37 °C in a 96-well plate as per the manufacturer’s instruction. Fluorescence was determined (excitation, 360 nm; emission, 460 nm) with a CytoFluor series 4000 plate reader (Applied Biosystems). Background fluorescence was determined in wells containing the assay buffer only.

### Data acquisition of human gliomas

Human gliomas used in this study were from The Cancer Genome Atlas (TCGA) or our institution as specified. The TCGA cohort contained 281 LGG and 160 GBM cases, of which the clinical information and RNA sequencing data were downloaded from the TCGA data portal (https://tcga-data.nci.nih.gov/tcga/) in July 2015. IDH mutation and 1p/19q codeletion status of LGG were taken from the dataset, as reported by TCGA Research Network [71]. IDH mutation status of GBM patients were determined by TCGA Somatic Mutation File. A total of 431 patients were included in this study (Supplementary table 3). AT/RT expression data were retrieved from published datasets [12] and analysed with GBM using HG133 microarray (GSE73038). We retrieved all the molecular AT/RT (histologically ATRT or PNET) and compared them with IDH wild-type GBM in this data set, and analysed then using GEO2R (https://www.ncbi.nlm.nih.gov/geo/info/geo2r.html).

### IHC staining

All IHC stainings were carried out on immunostaining instruments (Roche Ventana Discovery or LEICA BondMax) following manufacturer’s guidelines. The following antibodies were used in this study: anti-GPR158 (ab121388, Abcam), anti-ATRX (HPA001906, Sigma), anti-IDH1^R132H^ (DIA H09, Dianova), anti-Olig2 (ab33427, abcam), anti-Pdgfrα (ab15501, Abcam), anti-Sox2 (AB 5603, Chemicon) and anti-DCX (ab18732, Abcam).

### Image analysis

Histological slides were digitized on LEICA SCN400 scanner (LEICA, Milton Keynes UK) at 40× magnification. Digital image analysis was performed on Definiens Developer 2.4 (Munich, Germany). Image analysis was done as previously published [6].

## Electronic supplementary material

Supplementary Figure 1

Supplementary Figure 2

Supplementary Figure 3

Supplementary Data Table 1

Supplementary Data Table 2

Supplementary Data Table 3

Supplementary Data Table 4

Supplementary Data Table 5
