# Supplementary material for: Inhibition of GPR158 by microRNA-449a suppresses neural lineage of glioma stem/progenitor cells and correlates with higher glioma grades
Source: Oncogene. 2018 May 3;37(31):4313–33. doi: 10.1038/s41388-018-0277-1 (PMC6072706; doi:10.1038/s41388-018-0277-1)
Supplement: Supplementary file 1 — Supplementary Figure 1 [file 41388_2018_277_MOESM1_ESM.pdf]

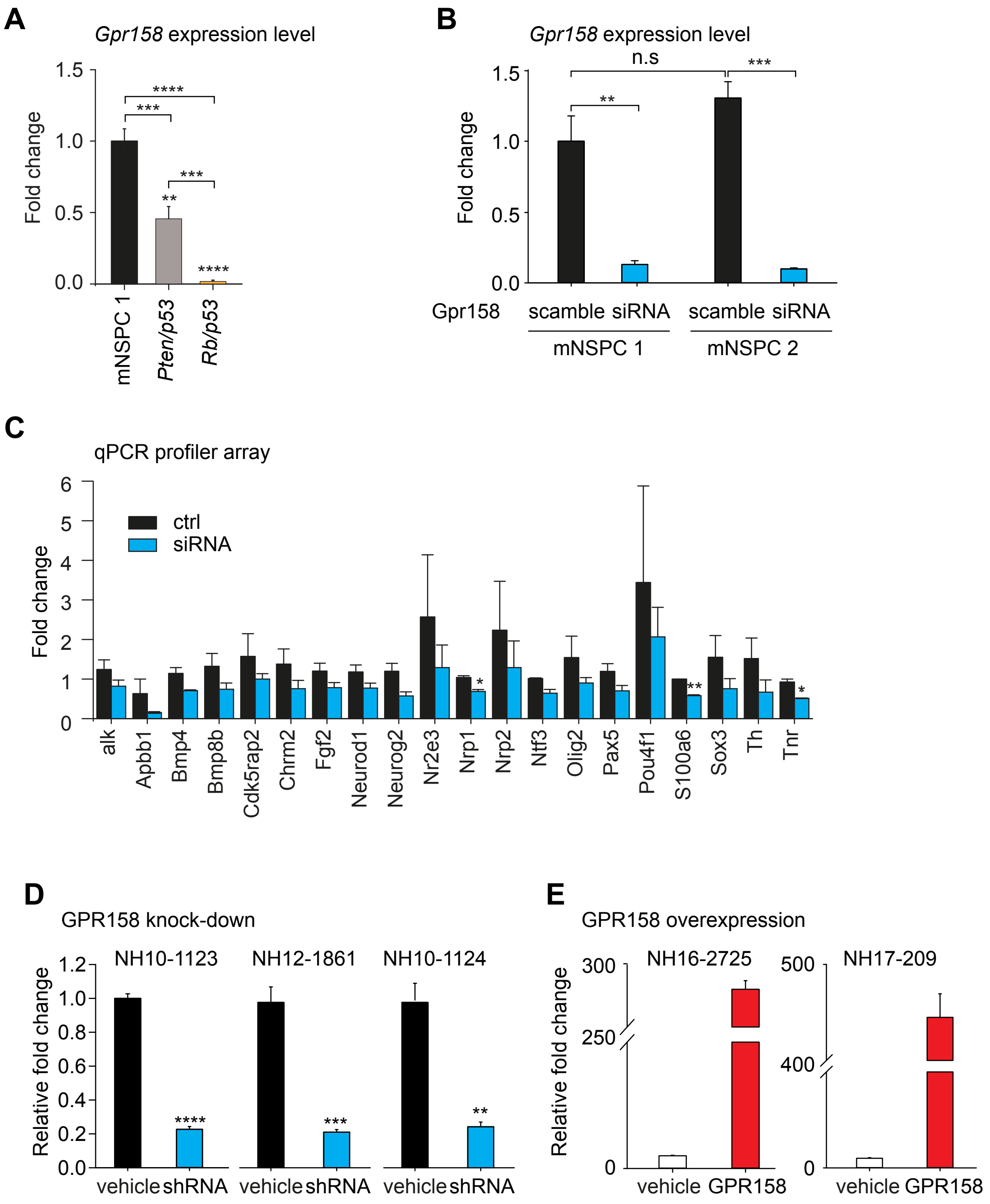
A
Gpr158 expression level
Fold change
mNSPC 1
Pten/p53
Rb/p53
B
Gpr158 expression level
n.s
Fold change
Gpr158
scamble siRNA
scamble siRNA
mNSPC 1
mNSPC 2
C
qPCR profiler array
ctrl
siRNA
Fold change
alk
Apbb1
Bmp4
Bmp8b
Cdk5rap2
Chrm2
Fgf2
Neurod1
Neurog2
Nr2e3
Nrp1
Nrp2
Ntf3
Olig2
Pax5
Pou4f1
S100a6
Sox3
Th
Tnr
D
GPR158 knock-down
NH10-1123
NH12-1861
NH10-1124
Relative fold change
vehicle shRNA
vehicle shRNA
vehicle shRNA
E
GPR158 overexpression
NH16-2725
NH17-209
Relative fold change
vehicle GPR158
vehicle GPR158
