# Supplementary figures and images for: Inhibition of GPR158 by microRNA-449a suppresses neural lineage of glioma stem/progenitor cells and correlates with higher glioma grades
Source: Oncogene. 2018 May 3;37(31):4313–33. doi: 10.1038/s41388-018-0277-1 (PMC6072706; doi:10.1038/s41388-018-0277-1)

## Forskolin/retinoic acid neuronal differentiation

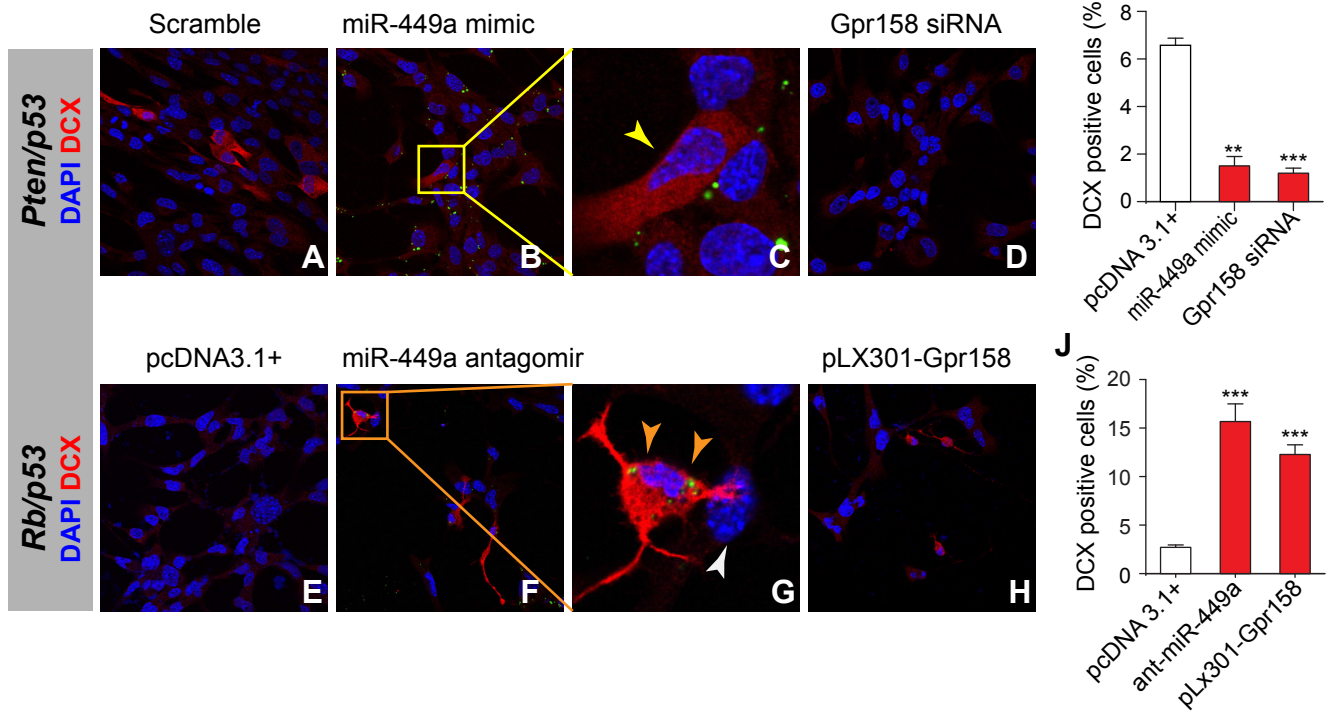

## LIF/BMP2 astrocytic differentiation

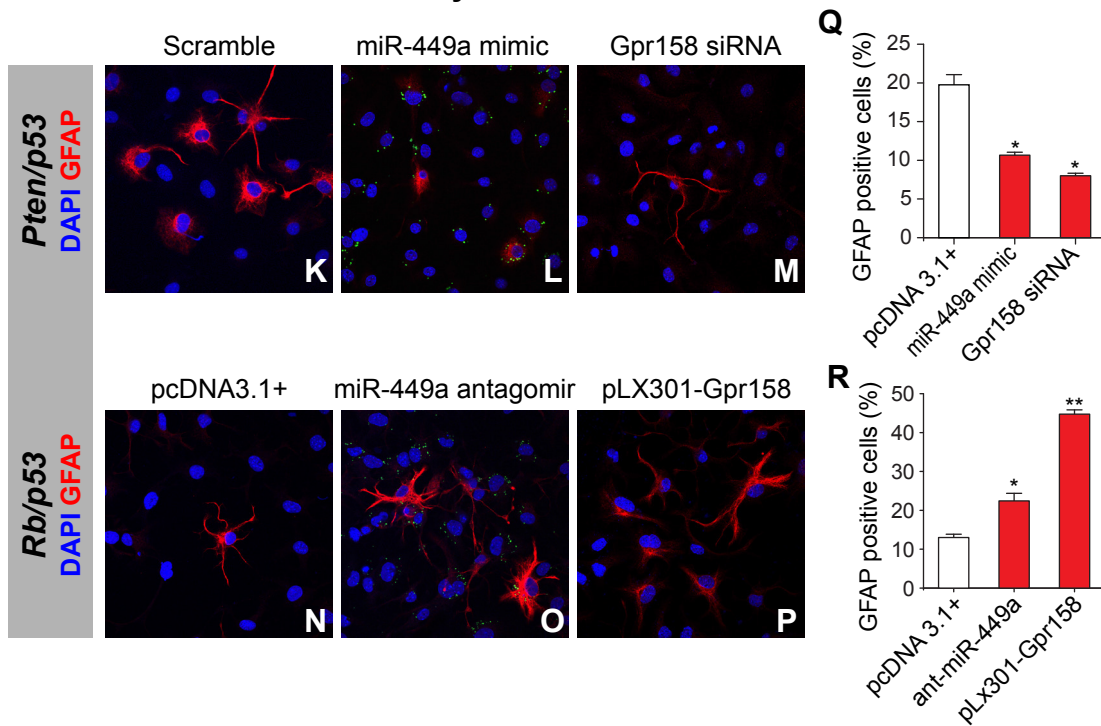

Supplement: Supplementary file 2 — Supplementary Figure 2 [file 41388_2018_277_MOESM2_ESM.pdf]

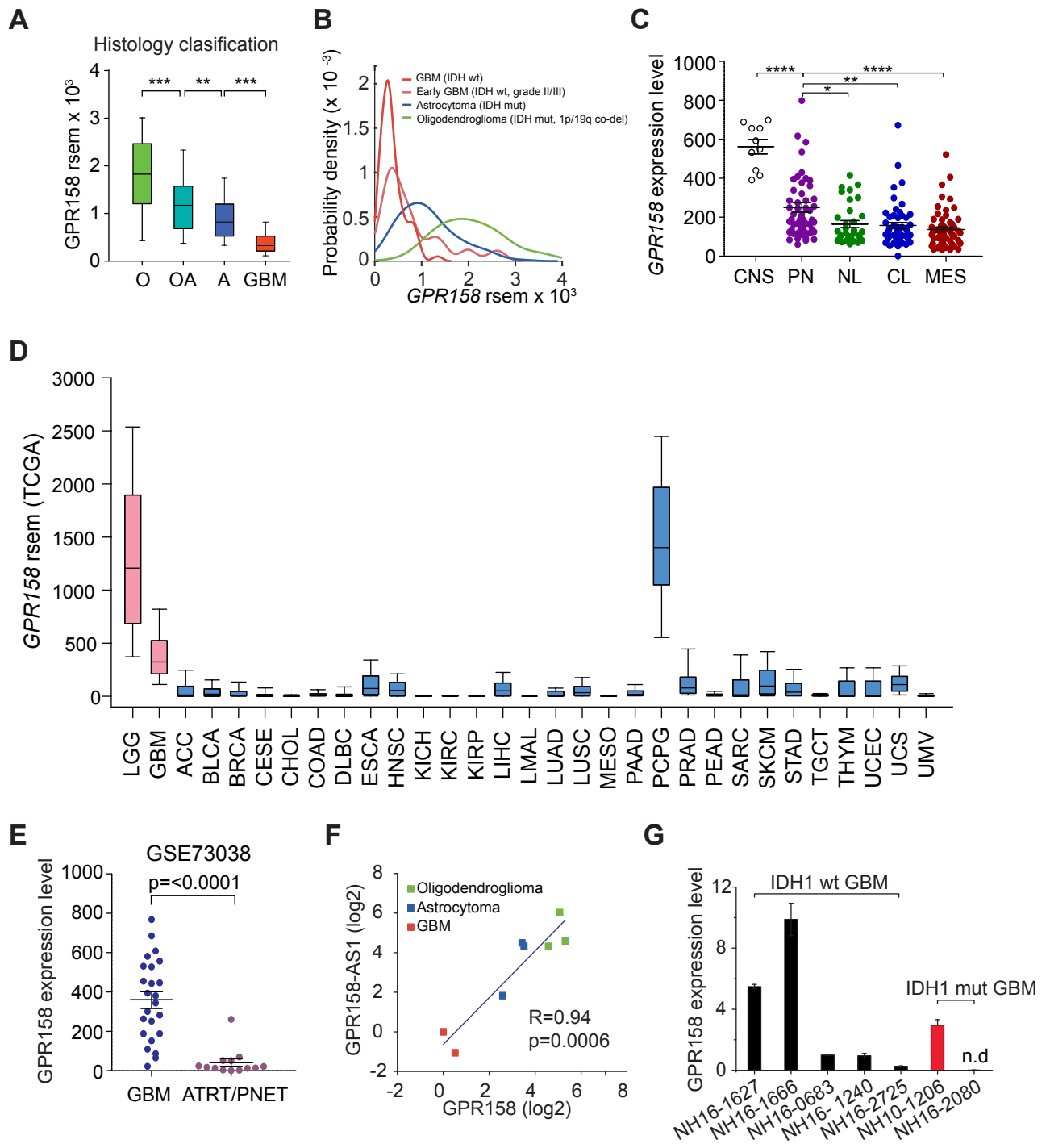

Supplement: Supplementary file 3 — Supplementary Figure 3 [file 41388_2018_277_MOESM3_ESM.pdf]
